# Efficacy and safety of Shen-Ling-Bai-Zhu-San combined with chemotherapy for lung cancer: A protocol for systematic review and meta-analysis

**DOI:** 10.1097/MD.0000000000024590

**Published:** 2021-02-12

**Authors:** Jiawang Jiang, Zhiming Li, Fenghao Zhang, Huaiyu Li, Renliang Li, Qianjie Qiu, Baoguo Chen

**Affiliations:** a Jiangxi University of Traditional Chinese Medicine; b The Affiliated Hospital of Jiangxi University of Traditional Chinese Medicine, Nanchang, Jiangxi Province, PR China.

**Keywords:** chemotherapy, lung cancer, protocol, Shen-Ling-Bai-Zhu-San, systematic review

## Abstract

**Background:**

Lung cancer (LC), with the high incidence in malignant tumors in the world, and seriously affects people's lives and brings a great economic burden. Previous clinical studies on Shen-Ling-Bai-Zhu-San (SLBZS) combined with chemotherapy for the treatment of lung cancer have been increasing, but there are no systematic reviews. This study aims to systematically study the efficacy and safety of SLBZS combined with chemotherapy in the treatment of LC.

**Methods:**

The Chinese and English databases will be searched by us for related documents, and the search time limit is January 2021. Databases including PubMed, Embase, Web of Science, the Cochrane Library, Chinese databases include China National Knowledge Infrastructure, Wanfang Data, ChongqingVIP Information Resource Integration Service Platform, China Biomedical Literature. The international clinical trial registration platform and the Chinese clinical trial registration platform will be searched by us to find ongoing or unpublished trials. After screening the literature based on inclusion and exclusion criteria, 2 researchers independently extracted data. The primary outcomes were the treatment efficiency. RevMan 5.3.5 software will be used for statistical analysis. The Recommendation, Assessment, Development, and Evaluation (GRADE) system will be used to evaluate the quality evidence of each result.

**Results:**

This study will provide the latest evidence for the SLBZS combined with chemotherapy for LC.

**Conclusion:**

The efficacy and safety of SLBZS combined with chemotherapy for LC will be evaluated.

**Unique INPLASY number:**

INPLASY202110025.

## 1 Introduction

Lung cancer (LC), also called primary bronchogenic carcinoma, is a malignant tumor that originates from the bronchial mucosa or glands.^[1]^ According to the statistical survey of the World Health Organization (WHO), lung cancer has become the main cause of cancer and 2.09 million lung cancer patients died worldwide in 2018.^[2]^ In addition, studies have found that lung cancer deaths account for 25% of all cancer deaths, and its 5-year survival rate is only 19%.^[3]^ Therefore, lung cancer has become a major public health problem that seriously threatens people and brought a huge financial burden all over the world.^[4]^

The surgery, radiotherapy, and chemotherapy are often used to treat lung cancer.^[5]^ The incidence of lung cancer is hidden, and it is difficult to find at an early stage. So it is already at an advanced stage when LC is diagnosed, and the opportunity for radical surgery has been missed.^[6]^ For the reason that chemotherapy has become the main treatment for LC.^[7,8]^ However, chemotherapy is often accompanied by adverse reactions such as bone marrow suppression, fatigue and diarrhea, which not only reduce the quality of life of patients, but also affect the course of treatment and even cause death.^[9–11]^ Based on this situation, there is an urgent to find a drug that can reduce the side effects of chemotherapy.

Modern studies have found that traditional Chinese medicine (TCM) as an adjuvant treatment for tumors can improve the efficacy of chemotherapy and reduce side effects, including Shen-Ling-Bai-Zhu-San (SLBZS).^[12,13]^ SLBZS is an ancient TCM formula and described in the “Tai Ping Hui Min He Ji Ju Fang “ in the Song Dynasty, which can treat cough, diarrhea, fatigue, and many other diseases in China.^[14]^ The SLBZS has clinical effects on the treatment of LC, which possible mechanisms of action include activation of PI3K-Akt-mTOR Signaling Pathway and inhibition of tumor growth promoters and antiapoptotic proteins enhances proapoptotic proteins.^[15,16]^ Moreover, SLBZS can adjust the microenvironment of the intestinal flora, improve the body's immunity, and ultimately increase the efficacy of chemotherapy and reduce the side effects.^[17,18]^

In summary, it is worth exploring whether SLBZS combined with chemotherapy is effective and safe for patients with LC. However, there is no evidence-based medicine research to support it. Therefore, it is necessary to evaluate the efficacy and safety of SLBZS combined with chemotherapy for LC.

## 2 Methods

### 2.1 Study protocol and registration

The protocol has been registered on the INPLASY website and registration number were INPLASY202110025 (URL https://inplasy.com/inplasy-2021–1–0025/). This study will be reported in accordance with the Preferred Reporting Project (PRISMA-P) Statement Guidelines for Systematic Reviews and Meta-Analysis Agreements.^[19]^

### 2.2 Study search

The English databases include PubMed, Embase, Web of Science, Cochrane Library, and Chinese databases include China National Knowledge Infrastructure (CNKI), Wanfang Data, Chongqing

VIP Information Resource Integration Service Platform (VIP), China Biomedical Literature (CBM) will be searched from the establishment of the database to January, 2021. The key words include “shenlingbaizhusan”, “chemotherapy”, “lung cancer”, “primary bronchogenic carcinoma”, “lung neoplasms”, “lung carcinoma” and “random allocation”. We will also retrieve ongoing or unpublished trials from the International Clinical Trial Registration Platform and Chinese Clinical Trial Registry Platform. PubMed's search strategy is shown in Table 1. These search terms will be accurately translated into other databases.

**Table 1:** Search strategy used in PubMed database.

Order	Search items
#1	(“Lung Neoplasms”[Mesh]) OR (((((((((((((((((Pulmonary Neoplasms[Title/Abstract]) OR (Neoplasms, Lung[Title/Abstract])) OR (Lung Neoplasm[Title/Abstract])) OR (Neoplasm, Lung[Title/Abstract])) OR (Neoplasms, Pulmonary[Title/Abstract])) OR (Neoplasm, Pulmonary[Title/Abstract])) OR (Pulmonary Neoplasm[Title/Abstract])) OR (Lung Cancer[Title/Abstract])) OR (Cancer, Lung[Title/Abstract])) OR (Cancers, Lung[Title/Abstract])) OR (Lung Cancers[Title/Abstract])) OR (Pulmonary Cancer[Title/Abstract])) OR (Cancer, Pulmonary[Title/Abstract])) OR (Cancers, Pulmonary[Title/Abstract])) OR (Pulmonary Cancers[Title/Abstract])) OR (Cancer of the Lung[Title/Abstract])) OR (Cancer of Lung[Title/Abstract]))
#2	(“Drug Therapy”[Mesh]) OR ((((((((((((((Therapy, Drug[Title/Abstract]) OR (Drug Therapies[Title/Abstract])) OR (Therapies, Drug[Title/Abstract])) OR (Chemotherapy[Title/Abstract])) OR (Chemotherapies[Title/Abstract])) OR (Pharmacotherapy[Title/Abstract])) OR (Pharmacotherapies[Title/Abstract])) OR (Vinorelbine[Title/Abstract])) OR (Cisplatin[Title/Abstract])) OR (Carboplatin[Title/Abstract])) OR (Gemcitabine[Title/Abstract])) OR (Docetaxel[Title/Abstract])) OR (Pemetrexed[Title/Abstract])) OR (Paclitaxel[Title/Abstract]))
#3	((((((shenlingbaizhu[Title/Abstract]) OR (SLBZS[Title/Abstract])) OR (shenlingbaizhusan[Title/Abstract])) OR (SLBZS[Title/Abstract])) OR (Chinese herbal medicine[Title/Abstract])) OR (traditional Chinese medicine[Title/Abstract])) OR (TCM[Title/Abstract])
#4	randomized controlled trial[Publication Type] OR randomized[Title/Abstract] OR placebo[Title/Abstract]
#5	#1 AND #2 AND #3 AND #4

### 2.3 Inclusion criteria for research selection

Studies that meet all of the following criteria will be included:

1.
*Type of studies.* This review will include randomized controlled trials (RCTs), whether using blinding.
2. *Type of participants.* Patients who are clearly diagnosed as lung cancer by pathological examination, and their pathological type and stage are not limited.
3. *Types of interventions.* The treatment group was treated with SLBZS (decoction, granule, tablet, and injection) combined with chemotherapy.
4. *Type of comparators.* The control group received conventional chemotherapy.
5. *Types of outcome measures.* The treatment efficiency (WHO curative effect standard for solid tumors)^[20]^ is the main outcome indicators of this study. The secondary indicators of this study include incidence of adverse reactions and improvement rate of quality of life.

### 2.4 Exclusion criteria for included studies

Studies that meet one of the following criteria will be excluded:

1. Republished literature.
2. Non-RCT literature.
3. Research data is incomplete or full text is not available.
4. Study on patients with other primary tumors except lung cancer.

### 2.5 Selection of studies and data extraction

**Figure 1:**
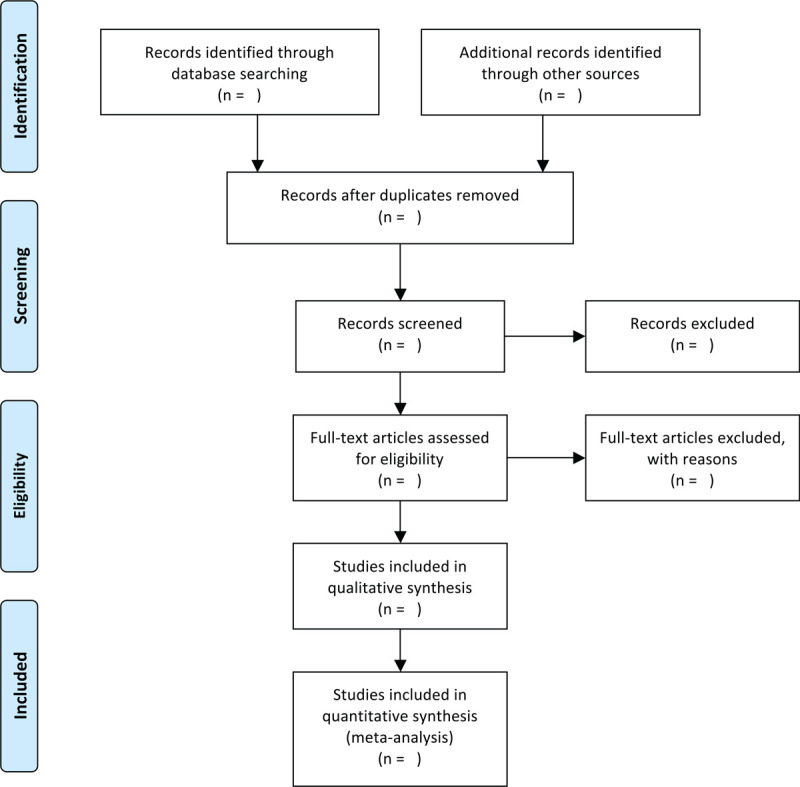
Flow chart of study selection.

Endnote20.0 software will be used by us to exclude duplicate studies. Two independent researchers will screen the literature after removing duplicates. Inconsistent opinions will be resolved through discussions with the third researcher. The selection process will be shown through the PRISMA flow chart (Fig. 1).

Two researchers independently extracted data based on predesigned forms and cross-checked them. The extracted content includes the first author, title, publication time, age, disease type, sample size, intervention measures, outcome indicators, and related data.

### 2.6 Risk of bias assessment

Methodological quality for each included trial will be assessed using the tools of Cochrane Handbook for Systematic Reviews of Interventions.^[21]^ The bias risk assessment category will include the following 7 areas: randomized sequence generation; allocation concealment; blinding of participants; blinding of outcome assessors; incomplete outcome data; selective outcome reporting; other bias. Two independent risk assessments of bias were conducted on the literature, and any differences would be discussed and resolved with the third researcher. Each assessment is labeled “high risk”, “low risk” or “unclear risk”.

### 2.7 Quantitative data synthesis and statistical methods

#### 2.7.1 Quantitative data synthesis

We will use Revman 5.3 software to conduct meta-analysis. If it is continuous data, it will be calculated based on the mean difference (MD) of the 95% confidence interval (CI), and the dichotomous data will be calculated based on the risk ratio (RR) of the 95% CI.

#### 2.7.2 Assessment of heterogeneity

Chi-Squared test and *I*^*2*^ test were used to test the heterogeneity of the included literature. When *P* > .1 and *I*^*2*^ < 50%, it indicates that there is no statistical heterogeneity between the studies; conversely, when *P* < .1 or *I*^*2*^ > 50%, it is considered that there is statistics heterogeneity between the studies.

#### 2.7.3 Assessment of reporting biases

Less than 10 studies will not be analyzed for reporting bias. If more than 10 studies are included, the symmetry of the funnel chart will be used to detect potential reporting bias.^[22]^

#### 2.7.4 Subgroup analysis and sensitivity analysis

We will conduct group analysis of chemotherapy regimen and pathological types of LC if possible. In order to evaluate the robustness of data analysis, sensitivity analysis will be performed.

#### 2.7.5 Grading the quality of evidence

The Recommendation, Assessment, Development, and Evaluation (GRADE) system will be used to evaluate the quality evidence of each result included, and the level of evidence will be divided into 4 levels: high, moderate, low, and very low.^[23]^ Therefore, it is necessary to use SLBZS that can increase efficacy and reduce toxicity and are low-cost to combine chemotherapy as an adjuvant treatment of lung cancer.

### 2.8 Ethics and dissemination

Because this study did not involve the personal data of participants, ethical approval is not required. The research will be published in a peer-reviewed journal or related journal meeting.

## 3 Discussion

At present, LC is a high-incidence disease in the world. Chemotherapy is currently one of the main treatments, which not only brings a huge economic burden, but also has many toxic side effects. SLBZS is a commonly used prescription for adjuvant treatment of lung cancer in China, which can reduce the financial burden of lung cancer patients because of low cost. Studies have shown that it can activate the PI3K-Akt-mTOR signaling pathway, adjust the microenvironment of the intestinal flora, and improve immunity.^[15,17,18]^ Te-Hsin Chao et al found that SLBZS can stimulate the secretion of ghrelin to increase food intake and has potential anti-tumor effects.^[24]^

RCTs have shown that SLBZS combined with chemotherapy for LC is safe and effective, but it has not yet been internationally recognized. Therefore, we hope that this study can provide the latest evidence for the effectiveness and safety of SLBZS combined with chemotherapy in the treatment of LC and guide clinical decision-making.

## Author contributions

**Conceptualization:** Jiawang Jiang and Zhiming Li.

**Data curation:** Jiawang Jiang, Fenghao Zhang, and Renliang Li.

**Formal analysis:** Qianjie Qiu, Huaiyu Li, Fenghao Zhang.

**Methodology:** Zhiming Li, Baoguo Chen, and Fenghao Zhang.

**Software:** Renliang Li, Huaiyu Li, and Jiawang Jiang.

**Supervision:** Jiawang Jiang and Zhiming Li.

**Writing – original draft:** Jiawang Jiang, Huaiyu Li, and Qianjie Qiu.

**Writing – review & editing:** Jiawang Jiang, Huaiyu Li, Renliang Li, and Qianjie Qiu.

## Abbreviations

Abbreviations: CIM = chemotherapy-induced myelosuppression, Cl = confidence interval, GRADE = Grades of Recommendation, Assessment, Development, and Evaluation, LC = lung cancer, MD = mean difference, PRISMA-P = Preferred Reporting Items for Systematic Review and Meta-Analysis Protocols, RCT = randomized clinical trial, RR = risk ratio, SLBZS = Shen-Ling-Bai-Zhu-San, TCM = traditional Chinese medicine, WHO = World Health Organization.
